# Comparison of the neonatal outcomes of progestin-primed ovarian stimulation and flexible GnRH antagonist protocols: a propensity score–matched cohort study

**DOI:** 10.3389/fendo.2023.1156620

**Published:** 2023-06-16

**Authors:** Mingze Du, Junwei Zhang, Bingnan Ren, Yichun Guan

**Affiliations:** The Reproductive Center, The Third Affiliated Hospital of Zhengzhou University, Zhengzhou, Henan, China

**Keywords:** progestin-primed ovarian stimulation, GnRH antagonist, preterm birth, low birth weight, small for gestational age, congenital malformation

## Abstract

**Objective:**

To compare the neonatal outcomes of progestin-primed ovarian stimulation (PPOS) and flexible gonadotropin-releasing hormone (GnRH) antagonist protocols.

**Methods:**

This was a retrospective propensity score–matched (PSM) cohort study. Women who underwent their first frozen embryo transfer (FET) cycle with freezing of all embryos followed by PPOS or GnRH antagonist protocols between January 2016 and January 2022 were included. Patients using PPOS were matched with the patients using GnRH antagonist at a 1:1 ratio. The main focus of this study was the neonatal outcomes of singleton live births, including preterm birth (PTB), low birth weight (LBW), small for gestational age (SGA), macrosomia and large for gestational age (LGA).

**Results:**

After 1:1 PSM, a total of 457 PPOS and 457 GnRH antagonist protocols were included for analysis. The average starting dose of gonadotropin (275.1 ± 68.1 vs. 249.3 ± 71.3, P<0.01) and total dose of gonadotropin (2799.6 ± 579.9 vs. 2634.4 ± 729.1, P<0.01) were significantly higher in the PPOS protocol than in the GnRH antagonist protocol. The other baseline and cycle characteristics were comparable between the two protocols. The rates of PTB (P=0.14), LBW (P=0.11), SGA (P=0.31), macrosomia (P=0.11) and LGA (P=0.49) did not differ significantly between the two groups. A total of 4 patients in the PPOS group and 3 patients in the GnRH antagonist group qualified as having congenital malformations.

**Conclusion:**

PPOS resulted in singleton neonatal outcomes similar to those of a GnRH antagonist protocol. The application of the PPOS protocol is a safe option for infertility patients.

## 1 Introduction

In the past 40 years, *in vitro* fertilization (IVF) has evolved rapidly, resulting in more than 5 million live infants worldwide (1, 2). The total number of IVF cycles currently exceeds 1 million per year, and the number of babies born exceeds 300,000/year in China (3). Compared with spontaneous pregnancy, conception by IVF is associated with a higher risk of adverse neonatal outcomes, including preterm birth (PTB), small for gestational age (SGA), low birth weight (LBW) and congenital malformation, even in singleton births (4–7). Controlled ovarian hyperstimulation (COH) is considered the key procedure of IVF, and the safety of its offspring has become a focus of attention.

Since the 1990s, Gonadotropin-releasing hormone (GnRH) antagonist protocols have been used to prevent a premature luteinizing hormone (LH) surge and are among the most widely used COH protocols (8, 9). The progestin prime ovarian stimulation (PPOS) protocol is a new COH protocol proposed by Kuang et al. in 2015 (10). In recent years, the PPOS protocol has been widely used in patients with polycystic ovary syndrome, normal ovarian response or low ovarian response, owing to its advantages in suppressing the LH surge as well as its oral route of administration (11–15). In our previous study, we analyzed the cumulative live birth rate of the PPOS and GnRH antagonist protocols, finding that the clinical efficacy of the two protocols was similar (15), but the safety of the offspring was unclear. Data comparing the neonatal outcomes between PPOS and GnRH antagonist protocols are scarce, and the type and dose of progesterone vary. Therefore, we designed a PSM cohort study to compare the singleton offspring outcomes between the PPOS and GnRH antagonist protocols to assess offspring safety with the PPOS protocol.

## 2 Materials and methods

### 2.1 Study design and population

This was a retrospective PSM cohort study performed at the Third Affiliated Hospital of Zhengzhou University. This study involved women who underwent their first frozen embryo transfer (FET)cycle with freezing all embryos followed by a PPOS protocol or a GnRH antagonist protocol between January 2016 and January 2022. This cohort study was approved by the review board of the Third Affiliated Hospital of Zhengzhou University. Eligible women were 20-42 years old and delivered singleton live births. Exclusion criteria were as follows: 1. Women with a history of recurrent spontaneous abortion, submucosal uterine fibroids, uterine malformations or endometrial polyps were excluded. 2.The maternal age is more than 42 years old or less than 20 years old. 3.Cycles were also excluded if the woman underwent preimplantation genetic testing (PGT) or if either partner had an abnormal karyotype. 4.Women were also excluded if they had chronic medical conditions associated with adverse perinatal outcomes, such as hypertension, diabetes, and severe liver disease. 5. The number of fetuses delivered was more than one.

### 2.2 Controlled ovarian hyperstimulation protocols

#### 2.2.1 Progestin-primed ovarian stimulation

The details of the operation of the PPOS protocol have been described in our previous study (15, 16). COH was initiated on the second or third day of the menstrual cycle. Depending on maternal age, body mass index (BMI), AMH and basal AFC, the starting dose of gonadotropin (Gn) was between 150 and 300 IU/day. Patients were administered 6 mg of medroxyprogesterone acetate (MPA) (Beijing Zhong Xin Pharmaceutical, China) on the same day as Gn. Then, follicle growth was monitored by vaginal ultrasound combined with serum hormone analysis 3-5 days later. If necessary, the dose of Gn was adjusted according to follicle development. When the diameter of the dominant follicle was greater than 20 mm or when at least three follicles reached 18 mm, the final stage of trigger ovulation was performed with triptorelin (100 μg) (Ferring International Center SA, Germany) and 2000 IU of human chorionic gonadotropin (hCG) (Lizhu Pharmaceutical Trading, China). Transvaginal ultrasound-guided oocyte collection was performed 36 hours later. Fertilization was carried out *in vitro* by IVF or ICSI, depending on the semen parameters.

#### 2.2.2 GnRH antagonist

Patients underwent a flexible GnRH antagonist protocol, described in detail in our previous study (15). In the flexible GnRH antagonist group, Gn (150 to 300 IU/day) was initiated on the second or third day of the menstrual cycle. The Gn dosage was adjusted based on the monitoring of follicle development by vaginal ultrasound combined with serum hormone levels. A GnRH antagonist at a dose of 0.25 mg/day was initiated once the diameter of the dominant follicle reached 12-14 mm and was continued up to the trigger day. The dosage of Gn was adjusted according to the follicle response. As soon as the diameter of the dominant follicle was greater than 20 mm or when at least three follicles reached 18 mm or when 50% of the dominant follicles reached 16 mm, ovulation induction was cotriggered with triptorelin (100 μg) (Ferring International Center SA, Germany) and 2000 IU hCG (Lizhu Pharmaceutical Trading, China). Oocyte retrieval was performed 36 hours later. Conventional IVF or ICSI was chosen based on the semen parameters.

### 2.3 Endometrial preparation protocols and frozen embryo transfer

In this study, all the embryos were vitrified. Frozen embryo transfer (FET) can be performed in the second menstrual cycle or later. Endometrial preparation protocols included the natural cycle, artificial cycle, induced ovulation cycle and downregulation + artificial cycle, which were mainly based on whether the patient’s ovulation was normal or not, and were also combined with the patient’s timing. The natural cycle was mainly used for women with regular menstrual cycles and spontaneous ovulation. Follicular development was monitored by transvaginal ultrasound, luteal-phase support was applied on the day of ovulation with oral dydrogesterone (2 times daily, 10 mg once) (Abbott Co. America) and intravaginal administration of 90 mg of a progesterone sustained-release vaginal gel (Merck Co. Germany), and 1-2 cleavage stage embryos were transferred 3 days later or 1 blastocyst was transferred 5 days later. The artificial/induced ovulation cycle for women with irregular menstrual cycles was done as previously reported (17). Downregulation + artificial protocols were used for women with endometriosis. GnRH agonist 3.75 mg was applied on the 2nd-3rd day of the menstrual cycle, and 28-30 days later, the endometrium was prepared with the artificial cycle. In women with clinical pregnancy, luteal-phase support was continued at least until 55 days after FET.

### 2.4 Outcome measures and definition

The primary concern of this study was neonatal outcomes. Small for gestational age (SGA) was defined as a birth weight less than the 10th centile for gestational age (18). Large for gestational age (LGA) was defined as a birth weight greater than the 90th centile of the sex-specific birth weight (18). The weight criteria refer to the weight of Chinese newborns (19). Other neonatal outcomes included preterm birth (PTB) (defined as a birth that took place after 28 weeks and before 37 completed weeks of gestational age), low birth weight (LBW, defined as a neonatal birth weight < 2500 g) (18) and macrosomia (defined as a neonatal birth weight ≥ 4000 g). We also analyzed neonatal malformation, defined as any condition so registered in the International Classification of Diseases Q codes, 10th Revision (ICD10:Q00–Q99) (20).

### 2.5 Statistical analysis

Statistical management and analyses were carried out using SPSS software, version 24.0. All data were obtained from the electronic medical record system of our reproductive center.

A PSM model was applied to balance the baseline characteristics between the PPOS and GnRH antagonist protocols, including maternal age; BMI; duration of infertility; type of infertility (primary/secondary infertility); infertility diagnosis (tube, male, male+female, others); basal serum FSH, AMH, and AFC levels; method of ART (IVF, ICSI); endometrial preparation protocols (natural cycles, artificial cycles, induced ovulation cycles, downregulation+artificial cycles); number of transferred embryos (1, 2); and type of transferred embryos (cleavage embryo, blastocyst). The propensity score was obtained from a logistic regression model. Patients given PPOS were matched with the patients given GnRH antagonist at a 1:1 ratio based on the propensity score with a standard caliper width of 0.2.

The normality of continuous data was checked by the one-sample Kolmogorov–Smirnov test. Continuous variables are shown as the mean ± SD, and Student’s t test was used to assess between-group differences. Categorical variables are represented as the number of cases (n) and percentage (%). The means from the chi-square test were used to assess the differences between groups with Fisher’s exact test when necessary. Two-sided P values of less than 0.05 were considered to indicate statistical significance.

## 3 Results

### 3.1 Study population

In total, 4023 women with singleton live births met the inclusion and exclusion criteria from January 2016 to January 2022 in our reproductive center. After applying the 1:1 PSM, a total of 457 PPOS and 457 GnRH antagonist protocols were included for analysis.

### 3.2 Baseline and cycle characteristics

**Table 1** reports the baseline characteristics of the patients included in the study. After the PSM model, the baseline characteristics, including maternal age (P=0.23), BMI (P=0.16), duration of infertility (P=0.64), type of infertility (P=0.11), infertility diagnosis (P=0.23), basal serum FSH level (P=0.41), AMH (P=0.24), AFC (P=0.13) and method of ART (P=0.78), were comparable between the PPOS and GnRH antagonist groups. The detailed reproductive outcomes of controlled ovarian hyperstimulation and the characteristics of the FET cycles are shown in **Table 2**. The average starting dose of Gn (275.1 ± 68.1vs. 249.3 ± 71.3, P<0.01) and the total dose of Gn (2799.6 ± 579.9 vs. 2634.4 ± 729.1, P<0.01) were significantly higher in the PPOS protocol than in the GnRH antagonist protocol. There were no statistically significant differences in the number of days of ovarian stimulation (P=0.052), number of oocytes retrieved (P=0.84), 2PN (P=0.98), available embryos on Day 3 (P=0.92), good-quality embryos on Day 3 (P=0.92), endometrial preparation protocols (P=0.07), endometrial thickness on the day of FET (P=0.21), number of transferred embryos (P=0.32) or type of transferred embryo (P=0.43) between the PPOS and GnRH antagonist protocols.

**Table 1:** Characteristics of the participants at baseline.

Characteristic	PPOS	GnRH antagonist	P value
No. of cases	457	457	
Maternal age (year)	34.0 ± 4.9	33.7 ± 4.9	0.23
Body mass index (kg/m2)	23.5 ± 2.9	23.8 ± 3.0	0.16
Duration of infertility (year)	3.7 ± 3.0	3.8 ± 2.9	0.64
Type of infertility (%)			0.11
Primary infertility	30.9(141/457)	35.9(164/457)	
Secondary infertility	69.1(316/457)	64.1(293/457)	
Infertility diagnosis (%)			0.23
Tubal factor	40.5(185/457)	34.8(159/457)	
Male factor	12.3(56/457)	15.8(72/457)	
Male+female factors	25.2(115/457)	25.6(117/457)	
Others	22.1(101/457)	23.9(109/457)	
Basal FSH(IU/L)	8.2 ± 3.2	8.0 ± 3.3	0.41
AMH(ng/ml)	1.8 ± 2.2	2.0 ± 2.2	0.24
Basal antral follicle count	10.7 ± 4.2	10.2 ± 4.5	0.13
Method of ART (%)			0.78
IVF	67.2(307/457)	68.1(311/457)	
ICSI	32.8(150/457)	31.9(146/457)	

Data are presented as mean ± SD for continuous variable and %(n/N) for categorical variable. PPOS, progestin-primed ovarian stimulation; FSH, follicle-stimulating hormone; AMH, anti-Müllerian hormone; ART, assisted reproductive technology; IVF, in vitro fertilization; ICSI, intracytoplasmic sperm injection.

**Table 2:** Outcomes of controlled ovarian hyperstimulation and characteristics of frozen embryo transfer cycle.

Characteristic	PPOS	GnRH antagonist	P value
No. of cases	457	457	
Starting dose of Gn (IU)	275.1 ± 68.1	249.3 ± 71.3	<0.01
No. of days of ovarian stimulation	9.5 ± 1.6	9.7 ± 1.9	0.052
Total dose of Gn (IU)	2799.6 ± 579.9	2634.4 ± 729.1	<0.01
No. of oocytes retrieved	8.4 ± 4.3	8.3 ± 4.7	0.84
No. of 2PN	5.9 ± 2.9	5.9 ± 3.5	0.98
No. of available embryos on day 3	4.9 ± 2.7	4.9 ± 3.2	0.92
No. of good-quality embryos on day 3	3.1 ± 2.2	3.1 ± 2.5	0.92
Endometrial preparation protocols			0.07
Natural cycles	42.0(192/457)	49.0(224/457)	
Artificial cycles	44.4(203/457)	36.5(167/457)	
Induced ovulation cycles	2.2(10/457)	3.3(15/457)	
Down-regulation + artificial cycles	11.4(52/457)	11.2(51/457)	
Endometrial thickness on the first day of progesterone administration(mm)	9.4 ± 1.4	9.5 ± 1.6	0.21
No. of transferred embryos			0.32
1	54.7(250/457)	58.0(265/457)	
2	45.3(207/457)	42.0(192/457)	
Type of transferred embryos			0.43
Cleavage embryo	47,7(218/457)	45,1(206/457)	
Blastocyst	52.3(239/457)	54.9(251/457)	

Data are presented as mean ± SD for continuous variable and %(n/N) for categorical variable. Gn, gonadotropin.

### 3.3 Neonatal outcomes

The average neonatal birthweight in the PPOS group was 3311.0 ± 569.3 g, which was comparable to 3365.8 ± 541.4 g in the GnRH antagonist protocol (P=0.14)(**Table 3**). There were no statistically significant differences in the gestational week between the two protocols (38.2 ± 1.8 vs. 38.3 ± 1.7, P=0.41) or the sex of the newborn (P=0.55). The rates of PTB (P=0.14), LBW (P=0.11), SGA (P=0.31), macrosomia (P=0.11) and LGA (P=0.49) did not differ significantly between the two groups. A total of 4 newborns (Q17.0 Accessory auricle-2, Q21.1 Atrial septal-1, Q21.0 Ventricular septal defect-1) in the PPOS protocol and 3 newborns (Q17.0 Accessory auricle-1, Q21.1 Atrial septal-1, Q21.0 Ventricular septal defect-1) in the GnRH antagonist protocol qualified as having malformations according to the International Classification of Diseases.

**Table 3:** Comparison of singleton neonatal outcome between the two groups.

Characteristic	PPOS	GnRH antagonist	P value
No. of cases	457	457	
Neonatal birthweight (g)	3311.0 ± 569.3	3365.8 ± 541.4	0.14
Gestational week (week)	38.2 ± 1.8	38.3 ± 1.7	0.41
Gender of newborn			0.55
Male	51.9(237/457)	53.8(246/457)	
Female	48.1(220/457)	46.2(211/457)	
Preterm birth	12.7(58/457)	9.6(44/457)	0.14
Low birthweight	6.6(30/457)	4.2(19/457)	0.11
Small for gestational age	4.6(21/457)	3.3(15/457)	0.31
Macrosomia	10.9(50/457)	7.9(36/457)	0.11
Large for gestational age	18.4(84/457)	16.6(76/457)	0.49
Malformations in total, n	4	3	–
Malformation type, n			
Q17.0 Accessory auricle	2	1	
Q21.0 Ventricular septal defect	1	1	
Q21.1 Atrial septal defect	1	1	

Data are presented as mean ± SD for continuous variable and %(n/N) for categorical variable.

## 4 Discussion

In this retrospective PSM cohort study, we provided evidence of no significant difference in singleton neonatal outcomes between the PPOS and GnRH antagonist protocols. The application of the PPOS protocol was a safe option for infertility patients.

Since the first PPOS protocol was reported by Kuang et al. (10), it has been reported that the PPOS protocol can effectively control over preovulatory LH levels and achieve satisfactory clinical outcomes (11–15, 21). However, there are few studies on the offspring safety of the PPOS protocol. We found five retrospective cohort studies on the offspring safety of PPOS, but they had differences in the progesterone drugs, doses, and control COH protocols (11, 22–25). Zhu et al. (22) compared the adverse neonatal outcomes of the PPOS protocol using progesterone soft capsules (brand name: Utrogestan) 200 mg daily with those of a short GnRH antagonist protocol in patients with FET. The neonatal outcomes, including PTB, LBW, gestational age, mode of delivery and live-birth defects, were comparable between the two groups. The retrospective cohort study of Wang et al., which further expanded the sample size (1589 live-born infants), showed that neonatal outcomes and the risk of congenital malformations were similar between the PPOS (Utrogestan) and short GnRH antagonist groups (1.52% vs. 1.63%) (23). Another retrospective cohort study, including 4596 live-born babies, found no significant differences in the overall incidence of congenital malformations between the PPOS (MPA 10 mg daily), GnRH antagonist protocol or mild ovarian stimulation (AOR=1.22, 95% CI= 0.61-2.44 and AOR=1.22, 95% CI=1.38 95% CI= 0.65-2.93) (24). A study performed in 2019, unlike previous studies, used dydrogesterone in the PPOS protocol. The results showed no significant differences in the rate of LBR, PTB, SGA or LGA after adjustment for confounding factors for both singletons and twins, suggesting that the application of the PPOS protocol using dydrogesterone (20 mg daily) was a safe option for newborns (11). Another retrospective cohort study included patients who had advanced endometriosis and assessed live-birth congenital malformations of PPOS (n=1203), GnRH agonist (n=221) and GnRH antagonist (n=71). The GnRH antagonist group (1.41%) and the GnRH antagonist protocol group (1.8%) had similar incidences of congenital malformations as the PPOS group (1.33%) (25).

In this study, unlike other studies, 6 mg daily MPA was given in the PPOS protocol. The range of MPA application is 4-10 mg in the PPOS protocol in our reproductive center, and 6 mg is most common. According to the study by Dong et al. (26), the number of oocytes retrieved and the pregnancy outcome of the PPOS protocol were similar to those of the standard protocol, suggesting that 4 mg of MPA daily was sufficient to prevent an LH surge in women undergoing IVF/ICSI treatment. However, there are currently no studies on the effect of different doses of MPA on offspring outcomes. One clinical study reported the neonatal outcomes of MPA at a dose of 10 mg per day (11, 25). Considering the most common MPA dose and to minimize the variability between results due to different drug doses, we included the PPOS protocol using 6 mg MPA. This study is the first clinical study to use a flexible GnRH antagonist protocol as the control group. On the one hand, GnRH antagonists have been widely used in the clinic since their introduction in the 1990s (8, 9). Many clinical studies have explored their efficacy and safety (27, 28). A large number of studies have confirmed the offspring safety of the GnRH antagonist protocol (29–31). Therefore, the GnRH antagonist protocol can be regarded as a standard protocol against which to compare the PPOS protocol on safety. On the other hand, the PPOS protocol is often used in people with low ovarian response in our reproductive center, and the GnRH antagonist protocol is also widely used in this group of people. Aiming for consistency of the basic characteristics of the population, we chose the GnRH antagonist protocol as the control protocol. In our previous clinical study, the cumulative live birth rate was similar between the PPOS and GnRH antagonist protocols in the low-ovarian-response population under the Poseidon criteria, but the safety of the offspring was not analyzed (15). While the PPOS protocol achieves satisfactory clinical outcomes, more consideration should be given to offspring and perinatal safety. The clinical data provided in this study illustrate the offspring safety of the PPOS protocol and provide evidence that can be used in the formulation of a clinical COH protocol.

This is the first PSM cohort study to analyze the neonatal outcomes of the PPOS protocol compared with those of the GnRH antagonist protocol. There are limitations to this study. First, there may be confounding factors due to the limitations of retrospective cohort studies. To reduce the influence of confounding factors on the observation indicators, we adopted strict inclusion and exclusion criteria and balanced the basic characteristics of the two groups through PSM. Second, this study mainly analyzed neonatal outcomes, including PTB, LBW, SGA, macrosomia, LGA and congenital malformation. Due to incomplete follow-up data, perinatal outcomes such as pregnancy complications were not analyzed, including gestational hypertension, diabetes, premature rupture of membranes, and placenta previa. We know that perinatal safety and reducing the incidence of complications during pregnancy are indicators for evaluating the safety of COH protocols. Therefore, in future studies, we will expand the sample size, extend the follow-up time, and further evaluate the effectiveness and safety of the PPOS protocol.

## 5 Conclusion

In this retrospective propensity score–matched cohort study, we provide evidence of no significant difference in singleton neonatal outcomes between the PPOS and GnRH protocols. The application of the PPOS protocol is a safe option for infertility patients. Our results can help in the formulation of a clinical COH protocol and can encourage additional clinical research and long-term follow-up studies of offspring.

## Data availability statement

The raw data supporting the conclusions of this article will be made available by the authors, without undue reservation.

## Ethics statement

The studies involving human participants were reviewed and approved by the Ethics Committee of the Third Affiliated Hospital of Zhengzhou University. Written informed consent for participation was not required for this study in accordance with the national legislation and the institutional requirements.

## Author contributions

MD and YG designed the study and selected the population to be included and excluded. BR and JZ performed the data extraction and analysis. YG reviewed the data. MD and JZ drafted this article. All authors contributed to the article and approved the submitted version. 
